# Normal parameters for diagnostic transcranial magnetic stimulation using a parabolic coil with biphasic pulse stimulation

**DOI:** 10.1186/s12883-022-02977-8

**Published:** 2022-12-31

**Authors:** Pimthong Jitsakulchaidej, Pakorn Wivatvongvana, Kittipong Kitisak

**Affiliations:** Department of Rehabilitation Medicine, Faculty of Medicine, Chiang Mai University, Chiang Mai, 50200 Thailand

**Keywords:** Diagnostic transcranial magnetic stimulation, Reference values, Normal participants, MT, MEP, CMCT, ICF, SICI, SP

## Abstract

**Background:**

TMS is being used to aid in the diagnosis of central nervous system (CNS) illnesses. It is useful in planning rehabilitation programs and setting appropriate goals for patients. We used a parabolic coil with biphasic pulse stimulation to find normal values for diagnostic TMS parameters.

**Objectives:**

1. To determine the normal motor threshold (MT), motor evoked potentials (MEP), central motor conduction time (CMCT), intracortical facilitation (ICF), short-interval intracortical inhibition (SICI), and silent period (SP) values. 2. To measure the MEP latencies of abductor pollicis brevis (APB) and extensor digitorum brevis (EDB) at various ages, heights, and arm and leg lengths.

**Study design:**

Descriptive Study.

**Setting:**

Department of Rehabilitation Medicine, Chiang Mai University, Thailand.

**Subjects:**

Forty-eight healthy participants volunteered for the study.

**Methods:**

All participants received a single diagnostic TMS using a parabolic coil with biphasic pulse stimulation on the left primary motor cortex (M1). All parameters: MT, MEP, CMCT, ICF, SICI, and SP were recorded through surface EMGs at the right APB and EDB. Outcome parameters were reported by the mean and standard deviation (SD) or median and interquartile range (IQR), according to data distribution. MEP latencies of APB and EDB were also measured at various ages, heights, and arm and leg lengths.

**Results:**

APB-MEP latencies at 120% and 140% MT were 21.77 ± 1.47 and 21.17 ± 1.44 ms. APB-CMCT at 120% and 140% MT were 7.81 ± 1.32 and 7.19 ± 1.21 ms. APB-MEP amplitudes at 120% and 140% MT were 1.04 (0.80–1.68) and 2.24 (1.47–3.52) mV. EDB-MEP latencies at 120% and 140% MT were 37.14 ± 2.85 and 36.46 ± 2.53 ms. EDB-CMCT at 120% and 140% MT were 14.33 ± 2.50 and 13.63 ± 2.57 ms. EDB-MEP amplitudes at 120% and 140% MT were 0.60 (0.38–0.98) and 0.95 (0.69–1.55) mV. ICF amplitudes of APB and EDB were 2.26 (1.61–3.49) and 1.26 (0.88–1.98) mV. SICI amplitudes of APB and EDB were 0.21 (0.13–0.51) and 0.18 (0.09–0.29) mV. MEP latencies of APB at 120% and 140% MT were different between heights < 160 cm and ≥ 160 cm (*p* < 0.001 and *p* < 0.001) and different between arm lengths < 65 and ≥ 65 cm (*p* = 0.022 and *p* = 0.002).

**Conclusion:**

We established diagnostic TMS measurements using a parabolic coil with a biphasic pulse configuration. EDB has a higher MT than APB. The 140/120 MEP ratio of APB and EDB is two-fold. The optimal MEP recording for APB is 120%, whereas EDB is 140% of MT. CMCT by the F-wave is more convenient and tolerable for patients. ICF provides a twofold increase in MEP amplitude. SICI provides a ¼-fold of MEP amplitude. SP from APB and EDB are 121.58 ± 21.50 and 181.01 ± 40.99 ms, respectively. Height and MEP latencies have a modest relationship, whereas height and arm length share a strong positive correlation.

## Introduction

The first TMS device was developed in 1985 by Barker et al. TMS stimulated the primary motor cortex (M1), causing muscles innervated by the stimulated corticospinal tract to contract [1, 2]. Since this report, TMS has been studied and applied to neurological disorders. Transcranial magnetic stimulation (TMS) uses the principle of electromagnetic induction based on Faraday’s laws to produce a designated effect on cortical excitability [2, 3]. An electrical current in a magnetic coil generates a magnetic field and induces an electrical current in corticospinal neurons. Suprathreshold stimulation generates action potentials, and then motor-evoked potentials (MEPs) are shown through surface electromyography (EMGs) on the target muscles [4, 5].

TMS stimulates the nerve at the neuronal level in the same way as traditional transcranial electrical stimulation (TES). The rapidly changing magnetic field induces an electrical current in the brain tissue. At suprathreshold stimulus intensities, the electrical current depolarizes cortical axons and causes action potentials [2]. TMS, unlike TES, can successfully excite cortical neurons while creating a low electrical current on the scalp. As a result, the patients tolerate it better [5].

Several descending corticospinal volleys may be detected in the cervical and lumbar spinal cord regions when a single-pulse TMS is applied to the M1. The initial volley, which has the smallest delay, may occur from direct activation of the fast-conducting corticospinal neuron at its axon hillock. The volley is known as a direct (D)-wave if it is triggered by a direct monosynaptic connection with the spinal motor neurons. Later, several waves project onto the pyramidal neurons as a result of indirect transsynaptic cortical spinal stimulation by separate sets of intracortical neurons. These volleys are referred to as indirect (I)-waves. The recruitment of the various components of the corticofugal discharge by a single TMS pulse is affected by the strength of stimulation, coil shape, pulse configuration, and direction of stimulation to produce current flow on each volley [5].

Six parameters are useful in diagnostic TMS [4, 5]. By using single-pulse TMS, we can gather motor threshold (MT), motor evoked potentials (MEP), central motor conduction time (CMCT), and silent period (SP). We can collect intracortical facilitation (ICF) and short-interval intracortical inhibition (SICI) using paired-pulse TMS.

MT is the action potential that propagates along the peripheral motor axons, inducing a motor response and is recorded with surface electrodes over the target muscle. By the relative frequency method as described by Rossini [6], this is the lowest TMS intensity required to elicit MEPs of more than 50 µV peak-to-peak amplitude in 5 out of 10 trials [2, 3, 7, 8]. MT is the first preference before other TMS measurements [3, 4, 7, 9]. For diagnosis purposes, we use MT coupling with others to evaluate the hypo/hyper-excitability of the hemisphere.

MEP resembles compound muscle action potentials (CMAP). We expected that the main parts of glutaminergic, NMDA, and AMPA receptors would play a major role in early indirect (I1) wave at lower intensities, but at higher intensities, the stimulation would be modulated by other monoamine transmitters, i.e., dopamine (DA), norepinephrine (NE), serotonin (5-HT), and acetylcholine (Ach) to act on late multiple I2-4 waves [10]. We used MEPs with a range of intensity from submaximal (120% MT) to supramaximal (140% MT) to elicit consistent MEPs with the least amount of pain for the patients. The stimulus–response curve was also investigated using the 140/120 MEP ratio, which revealed that the MEP response rises as TMS intensity increases [4, 7].

CMCT is defined as the latency difference between the MEPs induced by stimulation of the motor cortex and the MEPs induced by the spinal nerve root. Two methods can evaluate CMCT [7]. The first method is to apply stimulation to the spinal segments. The other method is using F-waves. Both methods have some limitations. However, in our setting, we found that using the F-wave method is simple and gives more comfort to the patients [11]. It is important to remember that the F-wave provides a shorter CMCT than direct spinal nerve root stimulation [4], which we will discuss later.

Silent period (SP) referred to as cortical SP is a period of electrical silence in the surface EMG activity that occurs immediately after the MEP during a tonic muscle contraction for a few hundred milliseconds [2]. The silent period is believed to be caused by inhibitory processes which are most likely mediated by GABA-B receptors [2, 3].

When using paired-pulse stimulation to assess intra-cortical inhibition and facilitation, there are two intensities of stimulus. The first stimulus is a subthreshold conditioning stimulus (CS), which uses 60–80% MT. The other stimulus is a suprathreshold tested stimulus (TS), which uses 120% MT. Two useful parameters are as follows. First, intracortical facilitation (ICF) occurs when a subthreshold CS is followed by a suprathreshold TS at an interstimulus interval (ISI) of 6–30 ms. ICF represents the excitatory glutamatergic function of M1. Second, short-interval intracortical inhibition (SICI) occurs when a subthreshold CS is followed by a suprathreshold TS at an ISI of 1–6 ms. SICI represents post-synaptic inhibition mediated by gamma-aminobutyric acid type A (GABA-A) receptors [2, 3, 7, 8].

At present, TMS helps diagnose central nervous system (CNS) diseases [2]. These include myelopathy [12], amyotrophic lateral sclerosis, multiple sclerosis [2], dementia [13], stroke [2, 14], neuropathic pain, Parkinson's disease, cerebellar ataxia, and dystonia [7]. Although the standard investigations for the diagnosis of CNS diseases are magnetic resonance imaging (MRI) and computerized tomography (CT), the prognosis of recovery cannot be evaluated by these imaging investigations. Diagnostic TMS can evaluate corticospinal tract integrity [2, 3], which is referred to as the prognosis of recovery in patients with CNS diseases [2]. Although the role of TMS in the study and modulation of metaplasticity in neurological disorders is still unclear [15, 16], it is helpful to plan rehabilitation programs and set the proper goals for the patients [2, 3, 17].

A different type of coil and stimulation pulse configuration might result in a different outcome. In the case of diagnostic TMS, a large circular coil is preferred over a figure-of-eight coil because of the simpler placement over the targeted M1 region, a bigger cortical volume, and better depth penetration, which is helpful for TMS of the M1-leg area. As a result, most research institutions use circular coils for diagnostic purposes [11, 18–21]. In our laboratory, we have found a parabolic coil more convenient. It is a circular coil in a parabolic shape that can contour to the shape of the scalp and has been debated to provide more power than other stimulation coil types [22].

The stimulus waveform is generally referred to as monophasic or biphasic. The monophasic has an initial effective neural activation, but the attenuated reverse current flow does not. Regardless of the more potent biphasic pulse configuration, in which the current direction is reversed twice, it is vital to note that these two wave patterns selectively activate different populations of neurons [5]. There is no consensus on the best guidelines for their use for diagnostic purposes. However, we discovered that monophasic configurations are more frequently employed for diagnostic reasons [11, 18–20] than biphasic pulse stimulation for treatment sessions [2, 4, 5, 7, 9]. However, we selected biphasic stimulation because it produces more powerful results, with the reversal phase lasting longer and being wider than the initial rising phase [2, 4, 5, 7, 9]. It is also possible to provide the therapy immediately after the diagnostic session.

The goal of this study was to determine the normal values for diagnostic TMS parameters such as MT, MEP, CMCT, ICF, SICI, and SP with parabolic coil and biphasic pulse configuration, which may differ from others. Also, we would like to address some diagnostic values that have not been reported in the previous literature. This normative data can be used to define a cut-off value that separates normal and abnormal measurements. Abnormal values can be justified as being 2.5 standard deviations (SD) away from the mean of the data [5, 11, 23].

## Material and methods

### Subjects

Healthy Thai participants aged 20–60 years old were recruited from Maharaj Nakorn Chiang Mai Hospital, Thailand from November 2018 to June 2019. The following were the criteria for exclusion [5, 19, 24]:

1. History of epilepsy or seizure within the past year
2. History of depression and screening for anxiety (HADS-A) > 7 and/or for depression (HADS-D) > 7 by the Hospital Anxiety and Depression Scale (HADS) [25].
3. History of CNS diseases or injuries such as strokes or traumatic brain injuries
4. Having an abnormal neurological examination (weakness, hyperreflexia, spasticity, or any positive long tract signs such as positive Babinski sign or Hoffman’s sign)
5. Having a skull abnormality, having a skull operation, or having intracranial metallic implants
6. History of cognitive impairment and screening by the Thai Mental State Examination (TMSE) ≤ 23 [26].
7. History of drug abuse or alcohol dependence
8. Having acute or chronic pain with a numeric rating scale > 4
9. Having neurostimulator implants such as vagal nerve stimulators, deep brain stimulators, stents, aneurysm clips/coils, cochlear implants
10. Having peripheral neuropathy symptoms such as numbness or carpal tunnel syndrome

### Sample size calculations

The sample size was computed using N4Studies V1.4.1. From the formula, based on data from Garassus [21], we used CMCT from TA by the F-wave method, which is 10.7 ± 1.77 ms. The calculated size was equal to 43. We expected 20% to drop out. Therefore, this study planned to study 50 participants.

$$n=\frac{{z}_{1-\frac{\alpha }{2}}^{2}{\sigma }^{2}}{{d}^{2}}$$

Z is the desired level of statistical significance (*p* < 0.05) = 1.96. σ is the standard deviation of the outcome variable from the previous study, which is 1.77. d is the acceptable standard error of the outcome, which is a 5% error from 10.7 = 0.535.

### Experimental designs

This study was a descriptive study design. All participants who passed the inclusion/exclusion criteria and had already signed the informed consent, received a single dose of diagnostic TMS. Before the intervention, blood pressure, heart rate, weight, height, arm, and leg length would be recorded. The arm length was measured from the right center of the axilla to the tip of the right middle finger while the patient was sitting in an upright position and fully abducted shoulder with elbow extension, wrist in a neutral position, and finger extension. The leg length was measured from the right anterior superior iliac spine (ASIS) to the right medial malleolus while the patient was supine. Any side effects that occurred during or after TMS would also be recorded. Participants could stop the intervention if they felt uncomfortable.

### Non-invasive brain stimulation (NIBS) intervention

Transcranial magnetic stimulation (TMS). The MagPro® R30 with Option is manufactured by MagVenture® A/S Lucernemarken, 15 DK-3520, Farum, Denmark.

We used the MMC-140-II Parabolic Coil with power control as provided by Magventure. The coil has a parabolic shape, which makes contouring with the scalp easier and provides powerful and focused stimulation [22]. 

A trained physiatrist gave TMS at the left M1. The surface EMG electrodes were placed on the right abductor pollicis brevis (APB) and the right extensor digitorum brevis (EDB) muscles, respectively. We searched for the motor threshold (MT) which indicated the minimum intensity of the stimulation that induced an MEP greater than or equal to 50 µV for 5 out of 10 stimulations (a 50% successive trial) [4, 9].

The landmark for stimulation was C3 (the left M1). The hand motor areas were always on the left side of the leg areas, which were likely close to or at the vertex. We divided half the line, which measured from Nasion (Ns) to Inion (In). Then, we divided half the line that was measured from both tragi to find the vertex. The C3 hand motor area was 5 cm to the left of the vertex [4, 5, 27].

### Parameters used in this study [4, 5].

#### Motor threshold (MT)

In our study, we selected the MT at rest instead of active MT to ensure that no alpha motoneuron was preactivated [4] and to avoid the hysteresis effect, i.e., the rightward shift of the stimulus–response curve [28]. We used the relative frequency methods to start from 30% of the maximal stimulator output (MSO), gradually increasing in steps by 5% and decreasing in steps by 1% MSO until TMS consistently evoked the MEPs as desired. We used this intensity as the resting MT (reported as % of MSO) [4–6].

#### Motor evoked potentials (MEP)

We used relaxed or resting MEPs as they mainly reflect the activation of low-threshold, small, and slowly propagating pyramidal tract neurons, which eliminate the interference from already-firing motor units at the cortical level at 120% and 140% MT (reported in a millisecond, ms as latency and millivolt, mV as amplitude). We measured MEP as peak-to-peak amplitude. We recorded 120% and 140% as it corresponds to the study stimulus–response relationship of corticospinal excitability as mentioned above. We employed the stimulus five times in a row at each intensity. The average of five recorded MEP graphs was utilized for data output. We reported latency as the mean and SD, amplitude as the median, and IQR due to the skewed nature of the data.

#### Central motor conduction time (CMCT)

This study used the F-waves method [2] to calculate CMCT (reported in a millisecond, ms). This method was more convenient and tolerable for the patients. The formula used is widely accepted in most previous research [2, 7].

$$CMCT\;=\;MEP\;latency\;-\;(F+M-1)/2\;$$

We used MEP latency at 120% MT. F is the shortest latency of the F-wave in the target muscle. M is the onset latency of CMAP in the target muscle.

#### Intracortical facilitation (ICF)

We selected the ICF, which is achieved by using the numbers of 80% [2] and 120% MT (the latter resembles MEP amplitude at 120% MSO) as CS and TS, respectively. The ISI, on the other hand, was arbitrarily set at 10 ms (reported in milliseconds, ms as latency and millivolts, mV as amplitude) [4]. As with MEP, we used five-time stimulation for the average value. We reported the ICF as the ratio of the MEP amplitude.

#### Short-interval intracortical inhibition (SICI)

By using the same intensities of stimuli as in ICF, however, ISI was set at 2 ms (reported in milliseconds, ms as latency and millivolts, mV as amplitude) [4]. We once again employed five-time stimulations for report data. We reported SICI as the ratio of the MEP amplitude [7].

#### Silent period (SP)

SP was recorded during a tonic muscle contraction of the APB and EDB. We measured SP as the time elapsed between the end of the stimulus interval (the end of MEP) and the recurrence of voluntary tonic EMG activity (reported in milliseconds, ms) [2, 4, 5]. We reported the SP from the average of the five times of stimulation as well.

**Fig. 1:**
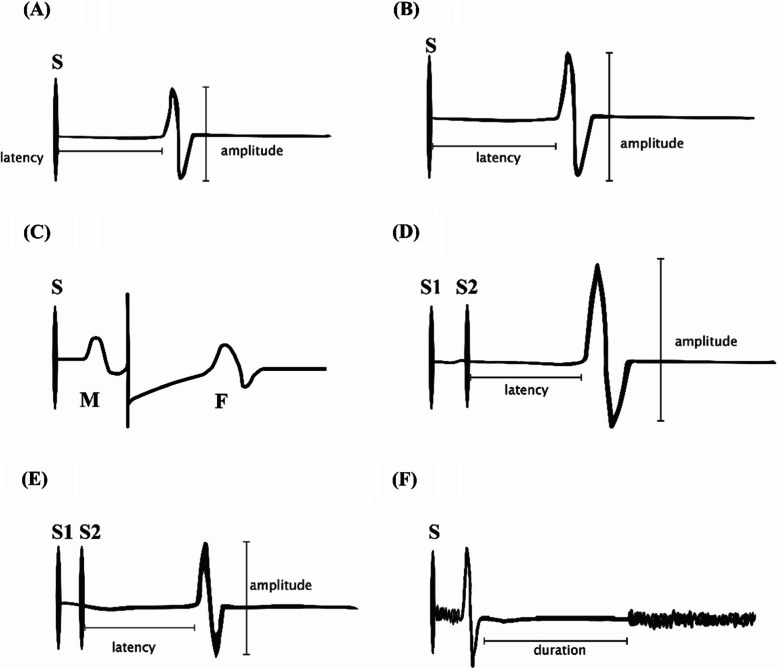
Waveform morphology of six parameters. (**A**) motor threshold; (**B**) motor evoked potentials; (**C**) F and M-wave (to calculate the central motor conduction time); (**D**) intracortical facilitation; (**E**) short-interval intracortical inhibition; (**F**) silent period; S = stimuli

Waveform morphologies and locations to measure latencies and amplitudes of all parameters are shown in Fig. 1.

All participants were given a single diagnostic TMS from 9 to 11 am on weekdays. They were advised to have a good sleep at night for at least 7 h and to refrain from drinking alcohol, coffee, tea, or any sedative drugs before the appointed date. Regular medicine and breakfast were taken as usual.

After the stimulation, immediate side effects such as hearing changes, headaches, scalp pain, and neck pain were noted and documented.

All methods were performed following the relevant guidelines and regulations by the Research Ethics Committee, Faculty of Medicine, Chiang Mai University.

### Data analysis

We used SPSS version 22.0 to analyze our data. The baseline characteristics of participants were evaluated. Outcome parameters were reported as mean and standard deviation (SD) or median and interquartile range (IQR), according to data distribution.

To compare the difference between MEP latencies and anthropometry, we used the mean of age, height, arm length, and leg length to divide our participants into two groups. The MEP latencies of APB and EDB between the two groups were evaluated using a Student’s t-test. The statistical significance level was set at *p* < 0.05.

## Results

### Subjects

**Fig. 2:**
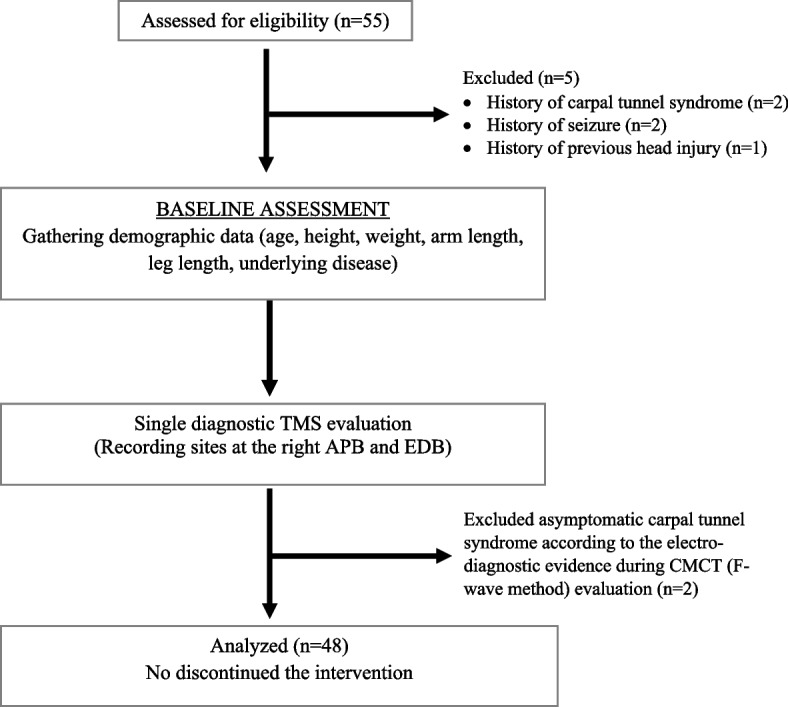
Research flow diagram

We screened a total of fifty-five healthy participants at Maharaj Nakorn Chiang Mai Hospital from November 2018 to June 2019. We excluded two participants with a history of carpal tunnel syndrome. We also excluded two participants with a history of seizures and one with a prior head injury. During the study, asymptomatic carpal tunnel syndrome from CMCT evaluation (prolonged distal latency of median nerve CMAP) was identified in two participants who were then excluded. Therefore, there were a total of forty-eight participants to analyze in our research, as shown in Fig. 2.

Baseline characteristics showed that all participants were equally distributed in each age group. They are both men, but mainly women. Weight, height, arm, and leg length were distributed in decrement patterns from a young age to an older age. As shown in Table 1, HADS-A, HADS-D, and TMSE showed normal cognitive function.

**Table 1:** General characteristics of the participants

	**Mean ± SD**
Age (years)	38.90 ± 12.03
Gender (Male/Female)	15/33
Weight (kg)	62.56 ± 15.26
Height (cm)	161.00 ± 7.64
Arm length (cm)	65.68 ± 4.01
Leg Length (cm)	84.62 ± 5.89
HADS-A	3.60 ± 1.99
HADS-D	1.90 ± 1.72
TMSE	28.27 ± 1.76

*Abbreviation*: *HADS-A* Hospital Anxiety and Depression Scale- Anxiety, *HADS-D* Hospital Anxiety and Depression Scale- Depression, *TMSE* Thai Mental State Examination

Outcome parameters were reported by mean and SD or median and IQR, according to data distribution. We used Shapiro–Wilk to test the normality of samples of less than 50. The null hypothesis is a normal distribution. If the test was significant (*p* < 0.05) and the distribution was skewed, then the median and IQR should be used. From our data analysis, latency parameters were normally distributed, so we used mean and SD. Amplitude and ratio were skewed, then we reported data by median and IQR.

To compare the difference in MEP latencies and anthropometry, we divided our participants into two groups based on age (< 40 and ≥ 40 years old), height (< 160 and ≥ 160 cm), arm length (< 65 and ≥ 65 cm), and leg length (< 85 and ≥ 85 cm).

### Diagnostic TMS parameters obtained from the right APB and EDB

MT and MEPs could be easily obtained from APB for all participants. Unlike APB, EDB could be recorded in only 30 of 48 participants (62% of the total), whereas the rest (38% of the normal participants) were not able to be elicited despite using the intensity of more than 80% MSO (most of the subjects also reported discomfort). All reported data is shown in Table 2.

**Table 2:** Normal parameters of the right APB and EDB

Parameters	APB (*n* = 48) Mean ± SD / Median (IQR)	EDB (*n* = 30) Mean ± SD / Median (IQR)
**MT (%)**	47.31 ± 8.06	60.37 ± 8.74
**MT**		
Latency (ms)	22.68 ± 2.02	38.76 ± 3.13
Amplitude (mV)	0.15 (0.11–0.25)	0.10 (0.07–0.23)
**MEP at 120% MT**		
Latency (ms)	21.77 ± 1.47	37.14 ± 2.85
Amplitude (mV)	1.04 (0.80–1.68)	0.60 (0.38–0.98)
**MEP at 140% MT**		
Latency (ms)	21.17 ± 1.44	36.46 ± 2.53
Amplitude (mV)	2.24 (1.47–3.52)	0.95 (0.69–1.55)
**140/120 MEP ratio**	1.92 (1.45–2.81)	1.74 (1.25–2.14)
**CMCT at 120% MT (ms)**	7.81 ± 1.32	14.33 ± 2.50
**CMCT at 140% MT (ms)**	7.19 ± 1.21	13.63 ± 2.57
**ICF**		
Latency (ms)	30.68 ± 2.27	44.93 ± 4.35
Amplitude (mV)	2.26 (1.61–3.49)	1.26 (0.88–1.98)
**ICF/120 MEP ratio**	2.18 (1.45–3.20)	1.70 (1.52–2.30)
**SICI**		
Latency (ms)	23.34 ± 1.98	39.04 ± 3.37
Amplitude (mV)	0.21 (0.13–0.51)	0.18 (0.09–0.29)
**SICI/120 MEP ratio**	0.24 (0.12–0.51)	0.24 (0.15–0.41)
**SP (ms)**	121.58 ± 21.50	181.01 ± 40.99

*MT* motor threshold, *MEP* motor evoked potentials, *140/120 MEP ratio* amplitude ratio of the MEP obtained at 140% and 120% MT, *CMCT* central motor conduction time, *ICF* intracortical facilitation, *SICI* short-interval intracortical inhibition, *SP* silent period, *IQR* interquartile range, latency values reported by Mean ± SD, amplitude and ratio values reported by Median (IQR), n of APB = 48, n of EDB = 30

### The difference between APB and EDB—MEP latencies in anthropometric measurements

Using data from diagnostic TMS parameters acquired from the right APB and EDB, we investigated MEP latency differences across age, height, and arm and leg length (shown in Table 3).

**Table 3:** The difference between APB and EDB—MEP latencies in anthropometric measurements

Parameters	MEP latency (ms)	Age < 40 years old	Age ≥ 40 years old	*p*-value
**APB**	**120% MT**	21.82 ± 1.54	21.71 ± 1.42	0.797
	**140% MT**	21.27 ± 1.56	21.05 ± 1.32	0.605
**EDB**	**120% MT**	37.46 ± 3.07	36.80 ± 2.67	0.543
	**140% MT**	36.55 ± 2.58	36.36 ± 2.57	0.841
		**Height < 160 cm**	**Height ≥ 160 cm**	***p-*value**
**APB**	**120% MT**	21.02 ± 1.25	22.45 ± 1.32	< 0.001*
	**140% MT**	20.33 ± 1.04	21.94 ± 1.33	< 0.001*
**EDB**	**120% MT**	36.46 ± 2.16	38.26 ± 3.56	0.150
	**140% MT**	35.93 ± 2.05	37.33 ± 3.07	0.152
		**Arm length < 65 cm**	**Arm length ≥ 65 cm**	***p*-value**
**APB**	**120% MT**	21.29 ± 1.31	22.24 ± 1.48	0.022*
	**140% MT**	20.56 ± 1.24	21.77 ± 1.39	0.002*
		**Leg length < 85 cm**	**Leg length ≥ 85 cm**	***p-*value**
**EDB**	**120% MT**	36.68 ± 2.24	38.02 ± 3.74	0.234
	**140% MT**	36.02 ± 2.24	37.29 ± 2.95	0.205

*MEP* motor evoked potentials, *APB* abductor pollicis brevis, *EDB* extensor digitorum brevis, latency values reported by Mean ± SD, *statistic significant (*p* < 0.05)

### Side effects of diagnostic TMS

Only three participants developed drowsiness, one had dizziness, and one had a mild headache after TMS sessions. All the TMS side effects disappeared in hours.

## Discussion

### Motor threshold (MT)

We measured that the MT of APB was 47.31 ± 8.06%. The value is slightly higher than data obtained from Rossini [4], 39–46%, and Triggs [20], 46.3 ± 7.2%. However, it is slightly lower than the MT recorded by Valls-Sole [29], 61.3 ± 9.6%. MT is variable in individuals for many reasons, such as relevant biological differences and sodium channel-blocking drugs that increase the resting MT [30]. The excitability of excitatory glutamatergic synapses, which connect the cortico-cortical fibers with the corticospinal neurons, also influences MT [31]. A more trivial factor relates to the inter-individual thickness of the convexity of the skull bones, which impacts the distance separating the stimulating coil from the excitable elements [32]. Another contributor is the number and density of cortico-cortical axons and corticospinal neurons in given target muscles [33].

The mean MT of EDB was 60.37 ± 8.74%. However, there is no reference to the mean MT of EDB in other studies. Therefore, we compare the MT of EDB in our study with that of other lower extremity muscles. We found that our data is at the lower normal limit but, corresponding to Rossini [4], the MT of TA was 60–80%.

We observed that the MT of EDB was higher than APB because the cortical homunculus of the foot is deeper than the hand [4, 5]. We always needed to increase coil intensity and adjust the coil medially and cephalad to the vertex from APB to EDB. However, we noticed that if we used MT with more than 80% MSO, most of the time patients felt uncomfortable, but no side effects were observed. Therefore, we had to cease the intervention before the MEP at EDB was elicited.

### Motor evoked potentials (MEP)

When we used the higher intensity, MEP latency was a bit shorter and the amplitude was much higher in comparison with MT, as shown in Table 2. However, the 120% MT could elicit an APB amplitude of MEP of more than 1 mV, which is accepted for use in most research [4, 5]. Therefore, we can use the APB latency and amplitude of MEP at 120% MT for diagnosis. However, be aware that 140% MT may provide better optimal conduction due to the larger amplitude of supramaximal stimulation. The median of MEP amplitude at 120% and 140% MT were 1.04 (0.80–1.68) and 2.24 (1.47–3.52) mV, respectively.

We usually observed higher MEP amplitude when increasing %MSO, which can be explained by stimulus–response curves [7] and also with preactivated muscle contraction. We found that MEP amplitudes obtained from previous studies use a high MSO, i.e., 70% and 85% [19], and 100% [18]. Some employed slight voluntary contraction, i.e., 15–25% of maximum [19], and more than 20% of maximum [21], which may be explained by alpha motoneuron preactivation [18]. However, we used the resting MEP with higher power requirements to reach the I3 wave and cause neuronal discharge [18]. This reason may explain why our MEP amplitudes were low between 1 and 2 mV but in an optimal range [2].

According to the study of the stimulus–response curve, TMS stimulus intensity and MEP amplitude share a relationship. This relationship shows a correspondence between MEP ranging from 120 to 140% MT [34]. Therefore, this stimulus–response relationship can be studied by calculation of the amplitude ratio of the MEPs obtained at 140% and 120% MT [4]. The ratio of 140/120 MEP would help us measure cortical excitability. The median 140/120 MEP ratio was 1.92 (1.45–2.81), which is nearly two-fold. If there is too much or too little deviation from this amount, one should be cautious of an abnormal response from the corticospinal network. Keep in mind that deprivation of sleep, caffeine, and other stress may aggravate many responses as well [35, 36].

In contrast to EDB, 120% MT could not elicit the MEP amplitude of EDB by more than 1 mV [0.60 (0.38–0.98) mV]. However, when we increased to 140% MT, the amplitude was nearly 1 mV [0.95 (0.69–1.55) mV]. As a result, the optimal intensity for diagnosis should be 140% of MT when recorded at EDB. The 140/120 MEP ratio of EDB is also nearly two-fold [1.74 (1.25–2.14) mV] as well, corresponding to the upper limb-APB recording.

However, in clinical practice, the single best-trial MEP with the largest amplitude is more suitable for use for analysis as this MEP reflects the optimal corticomotor conduction. Contrary to scientific TMS studies on corticospinal excitability, it may not be necessary to analyze or estimate the amplitude of all recorded MEPs for diagnostic TMS [5].

### Central motor conduction time (CMCT)

The method that we used for CMCT is the F-wave method. Our APB CMCT latency (7.81 ± 1.32 ms at 120% and 7.19 ± 1.21 ms at 140% MT) is nearly identical to Livingston [37], 7.8 ± 0.2 ms, and Eisen [19], 7.10 ± 2.0 ms, a tad longer than Furby [18] 6.1 ± 1.0 ms, and Claus [11] 5.8 ± 0.8 ms, and significantly longer than Rossini [38], 5.66 ± 0.84 ms in 16–35 years old and 5.45 ± 0.72 ms in 51–86 years old. CMCT discrepancies might be owing to differences in biological parameters, medicines, caffeine, sleep, and other external stress factors that may influence its outcome, regardless of coil type or stimulation power.

If there was no peripheral disease, CMCT calculated using the F-wave gives a shorter (1–1.5 ms) direct root stimulation with greater accuracy [4]. This could be due to the stimulation point to stimulate the nerve root being further away from the spinal motoneurons, e.g., 4 cm lateral to the spinal motoneurons [18]. The proximal root segment is therefore left between the cord and the exit foramen [7, 18]. As a result, the F-wave approach underrates CMCT and the longest F-wave should be employed [7]. However, both methodologies can provide the same results when it comes to estimating the place where motor fibers are depolarized by magnetic spinal stimulation [21].

The CMCT from EDB (14.33 ± 2.50 ms at 120% and 13.63 ± 2.57 ms at 140% MT) in our study are the same as Furby [18], 14.3 ms in males, and Stephan [39], 14.6 ± 2.9 ms. but slightly longer than Eisen [19], 13.1 ± 3.8 ms. Again, this disparity might be due to a variety of biological and environmental causes.

TMS penetration depth is restricted because dispersion increases exponentially with the proximity of the coil. TMS is effective at low stimulus levels in the M1-hand area, whereas greater intensities are required in the M1-leg area [5]. We calculated CMCT by using both 120% and 140% MT latencies. Both values were slightly different. According to optimal intensity, we propose utilizing 120% MT for APB and 140% MT for EDB. In other words, 120% MT is sufficient for the upper limb, whilst 140% MT is more suitable for the lower limb.

### Intracortical facilitation (ICF)

ICF represents the excitatory glutaminergic function in M1 by using CS and TS [2]. This value has not been previously reported. The paired-pulse stimulation with ISI at 10 ms can increase the amplitude abruptly by twofold of the MEP amplitude at 120% MT of both APB and EDB [ratio = 2.18 (1.45–3.20) and 1.70 (1.52–2.30), respectively]. This value helps to confirm the excitatory function of the cortex after single-pulse TMS. Furthermore, ICF helps to diagnose CNS diseases, such as patients with cerebellar diseases who have reduced ICF response. In contrast, patients with dystonia had an increased ICF response [7].

### Short-interval intracortical inhibition (SICI)

SICI is represented by post-synaptic inhibition mediated by GABA-A receptors [2]. Again, we are the first to report this value. The paired-pulse stimulation with ISI at 2 ms can decrease MEP amplitude at 120% MT for abruptly ¼-fold of both APB and EDB [ratios = 0.24 (0.12–0.51) and 0.24 (0.15–0.41), respectively]. These values help to evaluate the suppression function of cortical networks and CNS disease diagnosis. For example, patients with amyotrophic lateral sclerosis (ALS) or movement disorders have a reduced SICI response due to impaired inhibitory function [7]. Again, some machines could not produce paired-pulse stimulation.

Both ICF and SICI paired-pulse stimulations are useful in selecting the most appropriate medication for a patient by matching the identified abnormality of cortical facilitation or inhibition with the effects of various pharmaceutical agents [2]. In our setting, the paired-pulse stimulations, distinct from single-pulse, help physicians evaluate the hypo- or hyper-excitatory function of the hemisphere. Then, physicians can select the proper mode of therapeutic TMS for a patient based on cortical function.

As certain devices are incapable of delivering paired-pulse stimulation, other metrics from a single pulse, such as MT, MEP, 140/120 MEP ratio, and SP, cannot be directly substituted for this number because they indicate distinct electrophysiological processes and neurochemical consequences [40, 41].

### Silent period (SP)

The SP represents inhibitory mechanisms in the motor cortex which are most likely mediated by GABA-B receptors [2]. According to our findings, the SP from APB and EDB were 121.58 ± 21.50 and 181.01 ± 40.99 ms, respectively. These numbers are allied with Rossini [2, 4], whose SP from APB was approximately 100–300 ms. This suppression mechanism helps to confirm normal cortical excitability as well.

Furthermore, SP abnormality is beneficial for CNS diagnosis. For example, patients with acute stroke have a long duration of SP. In contrast, patients with ALS often have a short duration of SP due to intracortical inhibitory impairment [2]. This finding provides the pathophysiology of diseases and a treatment plan. Every TMS machine that has EMG monitoring could be able to record this value. However, we could not find any reference numbers from EDB. Thus, this is the first study to report the normal value of SP from EDB.

### The difference between APB and EDB—MEP latencies in anthropometric measurements

**Fig. 3:**
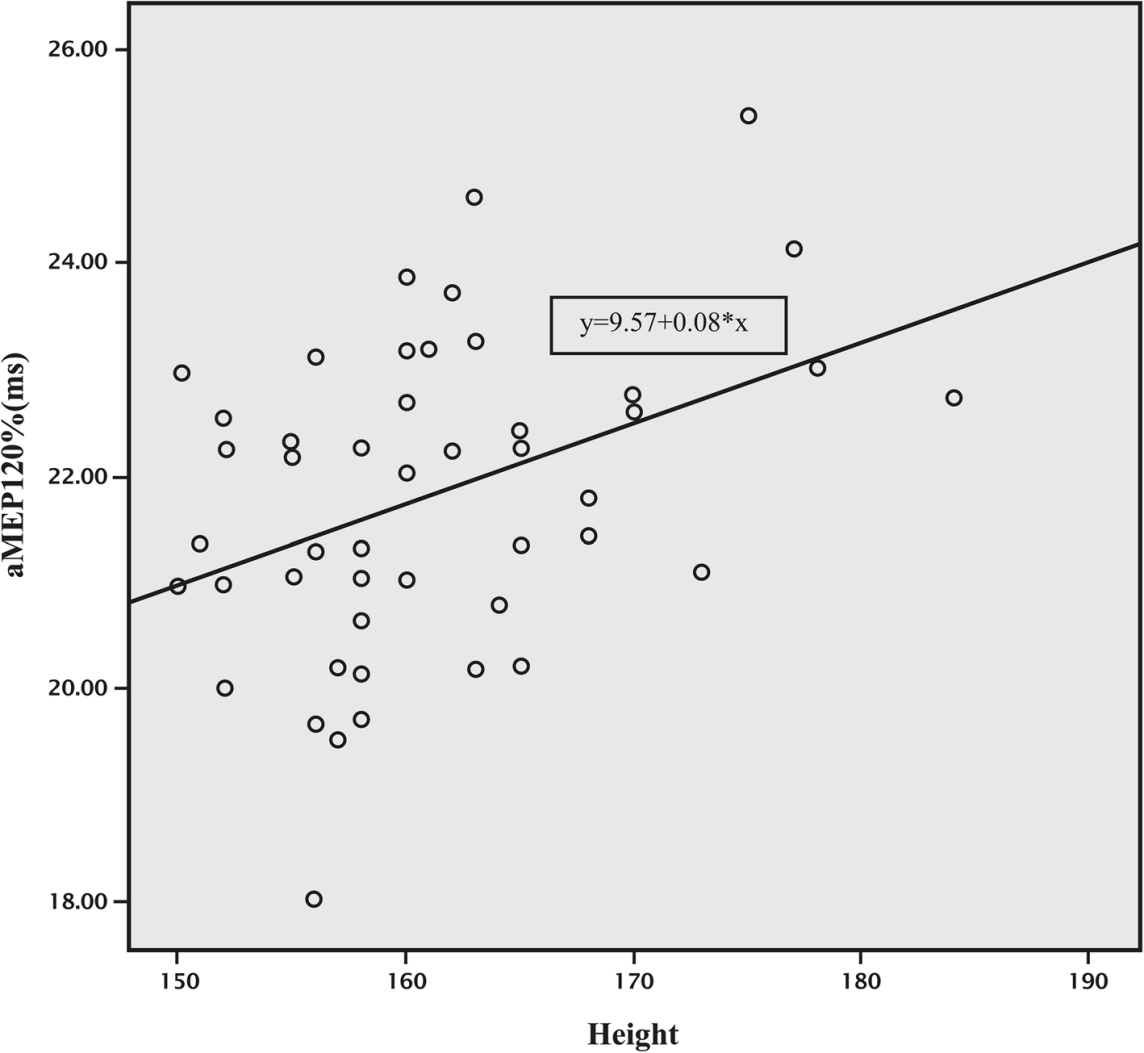
Correlation between height and APB latency of MEP at 120% MT. aMEP120% = APB latency of MEP at 120% MT, Correlation coefficient (r) = 0.379, APB latency of MEP at 120% MT = 9.57 + [0.08 x height (cm)]

**Fig. 4:**
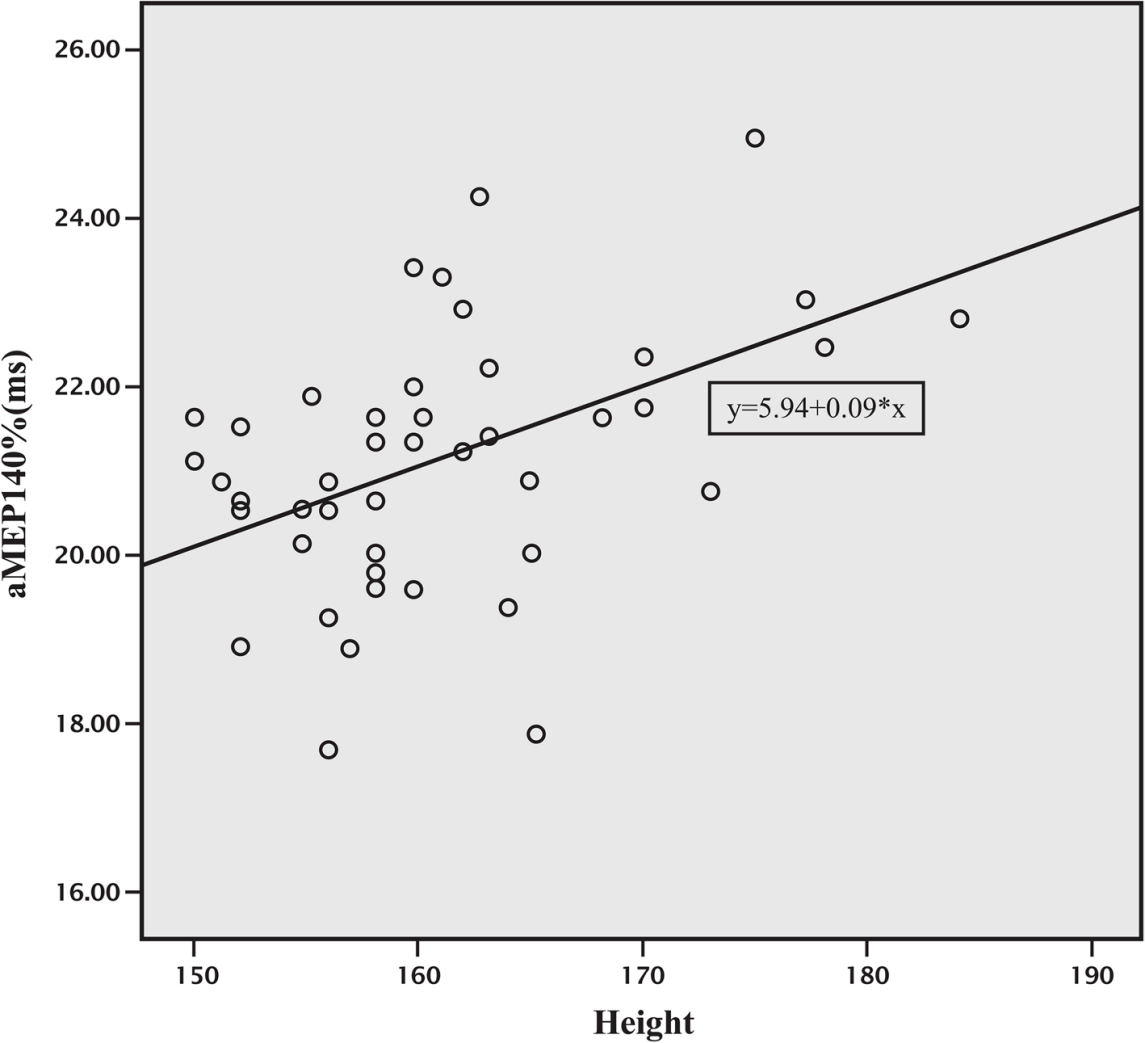
Correlation between height and APB latency of MEP at 140% MT. aMEP140% = APB latency of MEP at 140% MT, Correlation coefficient (r) = 0.509, APB latency of MEP at 140% MT = 5.94 + [0.09 x height (cm)]

We found a difference in MEP latencies of APB between height groups (< 160 and ≥ 160 cm) at 120% MT [21.02 ± 1.25 and 22.45 ± 1.32, t(46) = -3.86, *p* < 0.001], and also at 140% MT [20.33 ± 1.04 and 21.94 ± 1.33, t(46) = -4.65, *p* < 0.001]. The moderate correlation between height and MEP latencies of APB at 120% and 140% MT (*r* = 0.38 and 0.51, respectively) is shown in Figs. 3 and 4.

We also discovered only a difference in MEP latencies of APB between arm-length groups (< 65 and ≥ 65 cm) at 120% MT [21.29 ± 1.31 and 22.24 ± 1.48, t(46) = -2.36, *p* = 0.022] and 140% MT [20.56 ± 1.24 and 21.77 ± 1.39, t(46) = -3.20, *p* = 0.002]. However, there were no MEP latency differences in EDB between height groups and leg length groups. We could also elicit MEP of EDB for only 60% of participants. These findings are compatible with Eisen [19] who found a high relationship between arm length and thenar MEP latency (*r* = 0.65). But there was no correlation between TA latency and height. However, some studies found only a positive relationship between height and CMCT to the lumbosacral region. As a result, CMCT to upper limb muscles had no or only a slight relationship with height, but CMCT to lumbar segments had a substantial correlation with height [7, 11, 18]. These findings may suggest that the deeper the homunculus for the leg area, the more difficult it may be to elicit MEP of EDB. Also, the power may not be enough to show the relationship between MEP latencies of EDBs in height and leg length.

**Fig. 5:**
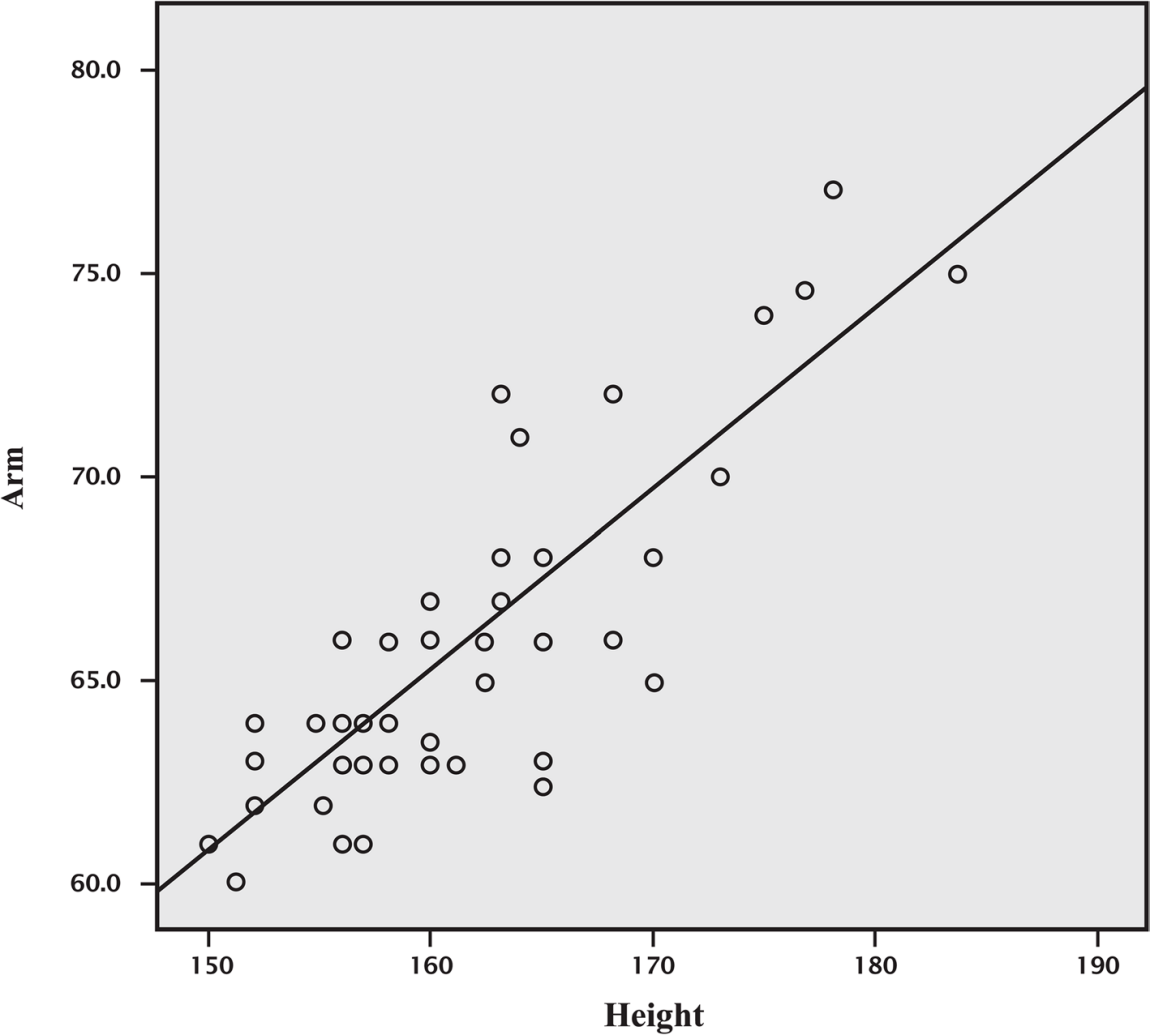
Correlation between height and arm length. Arm = Arm length, Correlation coefficient (r) = 0.756, Arm length (cm) = 5.11 + [0.44 x height (cm)]

As the height and arm length should be in linear proportion [42], we then compared the relationship between height and arm length and also found a high positive correlation between them (*r* = 0.756), as shown in Fig. 5. Therefore, we can select either height or arm length for anthropometric measurement to evaluate APB latencies.

There were no MEP latency differences between APB and EDB between age groups, as shown in Table 3. Age and sex have negligible effects on the MEP measures. The study of Groppa [5] also found that CMCT showed no significant age effect on stimulus intensity even if the intensity was high enough, unlike the cortical motor threshold and MEP amplitudes, which slightly changed with increasing age. This corresponds to the study by Chen [7], which found no or only a slight association with age.

Regarding sex, the study by Livingston [37], after adjusting MEP latencies to participants’ limb length, found no significant differences between males and females; only upper and lower limb lengths correlate with MEP latencies [7, 23]. However, there are only a few studies that contradict these results, which found a gender difference in the leg CMCT after controlling for differences in age and height [7, 23]. The reason for such a difference remains unclear and may be explained by the different methods used between the studies. Also, Akilan [43] attempted to explain the disparity between the RMT as measured by TMS and cognitive function, i.e., MMSE and RBANS scores. However, we found no difference in the mean RMT as 46.06 ± 7.81 in males and 49.96 ± 11.16 SD in females, which are in the normal range as mentioned in our study, 44.31 ± 8.06. Abnormal values are defined as being 2 or more conservatively as 2.5 standard deviations (SD) from the data's mean. At least this is negligible enough to interpret the difference between males and females.

### Side effects from diagnostic TMS

Unlike other therapeutic TMS reported in the last updated review [44], the side effects of diagnostic TMS seem to be less common. As allied to the study by Furby, there were no unwanted side effects from 50 participants [18]. Only a few participants in our study experienced tolerable transient side effects that resolved quickly after the TMS session. The reason may be explained by the amount and frequency of stimulation. Currently, diagnostic TMS uses only a few hundred pulses, which have a pause duration between each pulse for a few seconds, compared with a few thousand pulses with up to 50 pulses for theta-burst stimulation for a treatment session. Therefore, we can conclude that diagnostic TMS is safe and did not show serious side effects among the participants [3].

### Limitations of the study

EDB required more effort to elicit because the cortical homunculus of the foot is deeper than that of the hand. Only 30 participants could elicit an EDB. Therefore, this sample size might not be enough to show the effect of leg length on EDB latency. In future research, other muscles of the lower extremity might be selected for recording to find normal parameters. Also, it is cautioned that in the normal population, some individuals could not elicit the EDB.

Our participants were 20 to 60 years old. Therefore, the normal values of our study could not be compared with the parameters of patients less than 20 or more than 60 years old.

We chose the right APB and EDB from each participant as representative of the left corticospinal tract due to their ease of use and representation of the dominant side of the hemisphere. The left APB and EDB from the right cerebral hemisphere were not recorded in this study. Therefore, inter-side differences could not be assessed due to the lack of bilateral recording.

The rightward shift of the stimulus–response curve could be present due to the shorter duration of each stimulation [4]. We recognized that the longer duration, i.e., 20 s [28], is the best to ensure that the hysteresis effects would not have occurred. The shorter time interval may be suitable for the clinician in real-world practice.

## Conclusion

We established diagnostic TMS measurements using a parabolic coil with a biphasic pulse configuration. EDB has a greater MT than APB. The 140/120 MEP ratio of APB and EDB is two-fold. The optimal MEP recording for APB is 120%, whereas EDB is 140% of MT. CMCT by the F-wave is more convenient and tolerable for patients. ICF provides a twofold increase in MEP amplitude. SICI provides a ¼-fold MEP amplitude. SP from APB and EDB is 121.58 ± 21.50 and 181.01 ± 40.99 ms, respectively. Height and MEP latencies have a modest relationship, whereas height and arm length share a strong positive correlation.

## Data Availability

The original data from this study are available from the corresponding author upon reasonable request; all data generated or analyzed from this study are included in this published article.

## Abbreviations

TMS: Transcranial magnetic stimulation
MT: Motor threshold
MEP: Motor evoked potential
CMCT: Central motor conduction time
ICF: Intracortical facilitation
SICI: Short-interval intracortical inhibition
SP: Silent period
EMG: Electromyography
CMAP: Compound muscle action potential
CS: Conditioning stimulus
TS: Tested stimulus
ISI: Interstimulus interval
CNS: Central nervous system
ALS: Amyotrophic lateral sclerosis
GABA: Gamma-aminobutyric acid
APB: Abductor pollicis brevis
EDB: Extensor digitorum brevis
TA: Tibialis anterior
ASIS: Anterior superior iliac spine
HADS: Hospital Anxiety and Depression Scale
TMSE: Thai Mental State Examination
